# Book Notices

**Published:** 1868-01-01

**Authors:** 


### *BOOK NOTICES.*

---

Mechanical Therapeutics. A Practical Treatise on Surgical Apparatus, Appliances, and Elementary Operations, embracing Bandaging, Minor Surgery, Orthopraxy, and the Treatment of Fractures and Dislocations. By Philip S. Wales, M.D., Surgeon U.S.N. With 642 Illustrations. Philadelphia: Henry C. Lea. 1867.

A good book, and well calculated for the purpose it is intended to accomplish.

Surgeon Wales lays no claim to the making of a book "all out of his own head," but in this volume gives us an epitome of the practical experience of himself and others, in the briefest possible form, compatible with practical usefulness.

Part 1st Treats "of the Appliances of Dressing."

Part 2nd Treats "of Mechanical Bandages and Apparatus."

Part 3rd Treats "of Fractures, their Reduction, Dressings, and Apparatus."

Part 4th Treats "of Dislocations, their Reduction, Dressings, and Apparatus."

Part 5th Treats "of the Minor Operations in Surgery."

The illustrations are full, and the description of the form and mode of application of the numerous apparatus and appliances of the art of surgery so complete, that it seems to us that any one with an ordinary amount of surgical knowledge and mechanical skill may, with the aid of this volume, be able both to select and apply the proper apparatus, so as to secure all the benefits possible in any given case.

In our opinion, it is a good book, and one which every student and practitioner needs in his library. Especially would its value be appreciated by the surgeon whose field of practice is any wise remote from the larger cities, and where he has no full assortment of apparatus from which he may select in the treatment of particular cases.

Typographically, it is like all the books from this publisher—a success.

---

A Biennial Retrospect of Medicine and Surgery, and their Allied Sciences. Edited by Mr. H. Power and Dr. Austee, *et al.*, for the New Sydenham Society. Philadelphia: published by Lindsay & Blakiston.

---

On Diseases of the Lungs and Air Passages; their Pathology, Physical Diagnosis, Symptoms, and Treatment. By Henry William Fuller, M.D., Cantab., F.R.C.P., London, Physician to St. George's Hospital, etc., etc. From the Second and Revised London Edition. Philadelphia: Henry C. Lea, publisher.

The reputation of the first edition of this work is a sufficient guarantee of the value of this revised edition. Parts III. and IV., relative to diseases of the heart and great vessels, which made up a portion of the first, have been omitted in the second edition.

---

Compendium.—Among the very best of our exchanges, we count the *Reporter* of Philadelphia. Edited by S. W. Butler, M.D.

We notice with pleasure that the able and industrious editor has prepared a "Compendium," which is of the very highest order. It is the abstract and essence of the time, and we commend it strongly to the patronage of our readers. Having, unfortunately, mislaid the Prospectus, we are unable in this No. to give the terms, but will do so in our next. Meanwhile, we are content to advise our readers to address the Editor, S. W. Butler, M.D., 115 South Seventh Street, Philadelphia, enclosing, if we remember, $3, and receive in lieu one of the best works of the kind in the English language.

We will furnish the *Reporter* and this JOURNAL for $6 a year to advance-paying new subscribers, or the JOURNAL and "Compendium" for $5.

---

OPHTHALMIATRISCHE BEOBACHTUNGEN. Von DR. MED. ALBERT MOOREN, dirigirendem Arzt der Staedtischen Augen-Klinik zu Duesseldorf. Berlin, 1867. Verlag von August. Hirschwald, Unter den Linden, Nr. 68, pp. 345.

This volume is a report of the Clinical observations of Dr. Mooren in one of the richest Ophthalmic Clinics in Europe. It is a report of general results, rather than of special observations, and is arranged under sixteen chapters, as follows:

I. Clinical Statistics. II. Diseases of the Orbit. III. Diseases of the Lids. IV. Diseases of the Conjunctiva. V. Diseases of the Lachrymal Apparatus. VI. Diseases of the Cornea. VII. Diseases of the Sclerotic. VIII. Diseases of the Iris. IX. Diseases of the Choroid. X. Diseases of the Vitreous Body. XI. Diseases of the Lens. XII. Diseases of the Retina. XIII. Amblyopia. XIV. Amaurosis. XV. Disturbance of Accommodation. XVI. Diseases of the Muscles.

The scope and style of the work is novel, at least to us, and is highly illustrative of the great labor and satisfactory results of that labor, which has been performed in the wide field of Ophthalmology in the last few years. In no department of medicine have its devotees been more earnest than in this, and certainly in none have the results been more satisfactory.

Spermatorrhœa: Its causes, symptomatology, pathology, diagnosis, prognosis, and treatment. By Roberts Bartholow, A.M., M.D., Prof., etc., etc. Second edition, revised and augmented. New York: William Wood & Co., 61 Walker street. 1866.

---

Pennsylvania Hospital Reports, Vol. I. 1868. Philadelphia: Lindsay & Blakiston. Pp. 420.

---

Diseases of the Heart: Their diagnosis and treatment. By David Wooster, M.D., etc. San Francisco: H. H. Bancroft & Co. 1867. Pp. 216.

---

A Practical Treatise on the Diseases of Children. By D. Francis Condie, M.D., etc., etc. Sixth edition, revised and enlarged. Philadelphia: Henry C. Lea. 1868. Pp. 773.

---

Obstetric Clinic: A practical contribution to the study of Obstetrics and the Diseases of Women and Children. By Geo. T. Elliott, Jr., A.M., M.D., Prof., etc., etc. "*Plus on s'élève, plus l'horizon s'étend.*" New York: D. Appleton & Company, 443 and 445 Broadway. 1868. Pp. 458.

---

Hysteria: Remote causes of disease in general, treatment of disease by tonic agency, local or surgical forms of hysteria, etc. Six lectures delivered to the students of St. Bartholomew's Hospital, 1866. By F. C. Skey, F.R.S., etc., etc. New York: A. Simpson & Co. 1867. Pp. 103.

---

Annual Report of the Trustees and Superintendent of the Wisconsin State Hospital for the Insane, for the year ending September 30, 1867.

